# Medical Treatment of London Scholars

**Published:** 1921-03-26

**Authors:** 


### Medical Treatment of London Scholars.

### THE YEAR'S ARRANGEMENTS.

The L.C.C. Education Committee has prepared its scheme for the provision of medical and dental treatment of school children for the year commencing April 1 next. The work is to be extended and will involve an additional expenditure of £19,000, making the total over £90,000 a year. Special grants to centres are to be doubled, and grants of £100 will be made to enable voluntary committees to set up new centres. Both medical and dental staffs have asked for additional allowances, and medical officers will have their grants raised from £70 to £80 a year for service on one half-day a week, the dentists' fees being increased from the present rates of £50 and £55 a year for one half-day's weekly attendance to £65.

Regarding the payment to nursing associations, the allowance to local associations which supply nursing treatment was increased last year from £130 to £150 a year for the equivalent of one full-time nurse. This sum covered the cost of any drugs and dressings supplied in the children's homes and also the nurses' travelling expenses. As the Council's own nursing staff has had increased salaries the Committee proposes to increase the Association allowance to £175, but on condition that the increase shall be payable to the nurses.

Arrangements for dealing with eye defects will provide for 32,620 cases—an extra thousand—whilst the experiments in dealing with cases of squint will be continued.

Provision is to be made for 18,110 ear, nose, and throat cases, compared with 15,845, the increase being due to the refusal of some hospitals to allow school children to attend their aural departments. The class for the special treatment of mouth breathers will be continued and enlarged.

For minor ailments six new centres are proposed, so that a total of 53,350 cases can be dealt with.

Dental provision now covers the treatment of 107,360 cases and six more centres are scheduled for establishment.

For ringworm there will be arrangements for 2,628 cases. A department for *x*-ray treatment will be opened at the Queen's Hospital.

